# Technical Variations in Lateral Extra-Articular Tenodesis for Anterior Cruciate Ligament Reconstruction: A Systematic Review

**DOI:** 10.3390/jcm14186510

**Published:** 2025-09-16

**Authors:** Jan Zabrzyński, Bartosz Turoń, Adam Kwapisz, Achilles Boutsiadis, Maria Zabrzyńska, Maciej Sokołowski, Bartosz Majchrzak, Michalina Adamczyk, Katie Kellett, Gazi Huri

**Affiliations:** 1 Department of Orthopaedics and Traumatology, Faculty of Medicine, Collegium Medicum in Bydgoszcz, Nicolaus Copernicus University in Toruń, 85-092 Bydgoszcz, Poland; zabrzynski@gmail.com (J.Z.); sokolowskimaciej17@gmail.com (M.S.); bartoszmajchrzak97@gmail.com (B.M.); madamczyk01@o2.pl (M.A.); kellettkatie1@gmail.com (K.K.); 2 Department of Orthopaedics and Traumatology, Regional Hospital, 86-300 Grudziadz, Poland; b.turon@medipunkt.pl; 3 Department of Orthopedics and Pediatric Orthopedics, Medical University of Łódź, 90-419 Łódź, Poland; adam.kwapisz@gmail.com; 4 Military Hospital of Athens, 11525 Athens, Greece; boutsia@gmail.com; 5 Department of Family Medicine, Collegium Medicum, Nicolaus Copernicus University, 87-100 Torun, Poland; 6 Department of Orthopaedic and Sports Medicine, Hospital Doha, Doha P.O. Box 9958, Qatar; gazihuri@yahoo.com; 7 Department of Orthopaedics and Traumatology, Hacettepe University School of Medicine, 06100 Ankara, Turkey

**Keywords:** ACL surgery, LET technique, knee stabilization, the Lemaire-modified technique

## Abstract

**Background/Objectives**: The aim was to provide a comprehensive, systematic review on the Lateral Extra-articular Tenodesis (LET) methods used in anterior cruciate ligament (ACL) reconstruction in the light of recent data. **Methods**: To identify all of the essential studies that reported relevant data concerning primary outcomes: indications for surgery, surgical technique, graft type, fixation method, and tibial fixation location, an extensive search of the major and significant electronic databases (PubMed, Cochrane Central, ScienceDirect, Web of Science, Embase) was performed by three independent authors. A systematic investigation was conducted in November 2023, with no limits regarding the year of publication. After the database search, three independent reviewers screened all the papers, which followed strictly the inclusion and exclusion criteria, identifying a title, abstract, and full text concerning LET, surgical technique, femoral attachment, tibial attachment, graft type, fixation method, knee angle during fixation, and graft tension at fixation in ACL reconstruction. A systematic review of the collected literature was carried out according to the guidelines of Preferred Reporting Items for Systematic Reviews and Meta-Analyses (PRISMA). Study quality was assessed using the Cochrane Risk of Bias Tool. **Results:** Of the 35 papers reviewed, seven surgical techniques of LET differing in the way the procedure was performed were separated. The majority of papers were from Italy (n = 11), USA (n = 3), France (n = 3), and Canada (n = 3). The number of total participants across all studies was 6253. The majority of studies (17 papers) used the Lemaire modified procedure, and 10 papers used the MacIntosh technique modified by the Coker–Arnold approach. Most of the papers mentioned fixation location on the lateral distal part of the femur including six articles referring directly to lateral femoral epicondyle. Most authors (25 papers) defined tibial attachment as Gerdy’s tubercle. The most common graft was the iliotibial band and fixation method was sutures. The types of fixation in the surgical techniques of the collected papers were Sutures, Staples, Anchor, Interference screw, K-wire, Bioabsorbable Screw and Titanium Screw with a serrated polyethylene washer. **Conclusions:** Despite variability in technique, the Lemaire-modified procedure emerged as the preferred approach for Lateral Extra-articular Tenodesis, suggesting a general consensus around its reliability and reproducibility in clinical practice. The frequent use of the iliotibial band as graft material reflects its accessibility and suitability for reinforcing anterolateral stability. Similarly, the consistent use of sutures and fixation at Gerdy’s tubercle may indicate a favorable balance between technical ease and biomechanical strength. The variability in femoral fixation points—either at the lateral femoral condyle or epicondyle—highlights the ongoing debate or surgeon preference, underscoring the need for further comparative studies to establish optimal fixation strategy. Collectively, these patterns may help guide surgical decision-making, particularly when tailoring procedures to individual patient anatomy or surgical expertise.

## 1. Introduction

Anterior cruciate ligament (ACL) is one of the key structures that provide knee stabilization [1,2]. Disruption of the ACL is functionally disabling and may lead to consecutive injuries and increased risk of osteoarthritis, highly reducing the efficiency of movement [3]. The ACL injury is not only a sole tear of ligament, but a complex injury to the knee joint, including cartilage, anterolateral structures injury with avulsion Segond fracture of the lateral tibial plateau [4].

Recently, for the first time in history, the anatomy and role of the anterolateral ligament (ALL) has been described. The ALL is attached to the lateral femoral epicondyle anterior to the lateral collateral ligament (LCL) and lateral and posterior to Gerdy’s tubercle [5,6]. Studies regarding the ALL allow us to better understand the significance of this ligament in the biomechanics of the knee and change the perspective regarding procedures restoring its function, especially after ACL injuries [7,8,9].

The goal is to restore full stability of the knee joint and complete biomechanics, including all the injured structures. Isolated ACL reconstructive surgery not always gives good clinical outcomes with complete knee stability, and learning from anatomy, in some cases, combining ACLR and Lateral Extra-articular Tenodesis (LET) or ALL reconstruction, as a complex procedure, seems to provide a better outcome and return of the knee function compared with stand-alone procedures [10]. Which of the mentioned procedures is superior is still unclear.

The beginnings of the LET procedure in ACL injuries date back to the mid-1960s and are linked with Lemaire who introduced it as an isolated extra-articular procedure. However, later, LET lost its significance giving way to rapidly developing arthroscopic techniques, resulting in many surgeons abandoning this technique for decades [11]. The goal of anterior cruciate ligament reconstruction (ACLR) is to restore regular knee biomechanics, while LET is designed to additionally reduce ACL deficiency [12,13]. In light of recent studies, the topic of LET is being reviewed again, indicating that it is in fact a valuable and important additional technique suitable for patients with anterior cruciate ligament injuries and high instability of the knee [5,14,15]. Biomechanically, LET ensures transfer of loads from the ACL graft reducing the risk of its failure [2]. ACLR augmented with LET enforces control over the internal rotation of the tibia and reduces the pivot shift without decreasing the range of motion in flexion and extension [9]. This can lead to better results among those facing an increased risk of persistent instability after ACLR, most importantly in groups of patients such as young age, increased tibial slope, high-grade preoperative knee laxity, and athletes, the last group being the most susceptible to ACL injuries while also expecting an early return to sport [14,15].

Various LET operative methods have been presented through the years, allowing us to analyze and compare different aspects of the procedure [5]. The aim of this systematic review was to systematically review and compare the technical variations in LET procedures used in conjunction with ACLR, including graft type, path, fixation method, and anatomical landmarks. Secondary objectives were the evaluation of biomechanical and clinical outcomes associated with each LET technique, identifying the patient subgroups who may benefit most from LET-augmented ACLR.

The existing literature, while extensive, is inadequate for clinical decision-making due to its variability in surgical technique, inconsistent patient populations, and lack of long-term, high-quality comparative data. Many studies are retrospective or have limited follow-up, making it difficult to assess durability, risk of over-constraint, and long-term joint health. Moreover, the evolution of ACL graft types and rehabilitation protocols further clouds the direct applicability of earlier findings.

This is the first systematic review to categorize and compare the diverse surgical techniques of LET in ACL reconstruction, distinguishing it from prior SRMAs focused solely on clinical outcomes.

Standardizing LET techniques and terminology would improve the reliability and reproducibility of outcomes, enabling surgeons to make better-informed decisions tailored to individual patient profiles. It would also facilitate more rigorous and comparable clinical trials, and thus improve the quality of evidence and accelerating innovation in knee stabilization strategies.

LET, historically rooted in the pioneering efforts of Lemaire, MacIntosh, and others, has experienced a resurgence due to growing recognition of its biomechanical value in controlling anterolateral rotational instability. Its modern refinement, in combination with ACLR, demonstrates LET’s enduring relevance and its evolving role in optimizing outcomes for high-risk populations, such as young athletes or patients with high-grade pivot shift. Therefore, LET should not only be regarded as a foundational surgical method of the past but also a critical and adaptable technique for the future of knee ligament surgery.

We hypothesized that significant technical variation exists among LET procedures in terms of graft source, route, and fixation, and these differences influence clinical and biomechanical outcomes. LET techniques that utilize femoral fixation proximal and posterior to the lateral epicondyle and apply graft tension in extension provide better control of internal tibial rotation and pivot shift. Patients with defined risk factors (e.g., high-grade pivot shift, young age, increased tibial slope) achieve greater benefit from LET-augmented ACLR compared to standard ACLR alone.

## 2. Materials and Methods

### 2.1. Search Strategy

**Figure 1:**
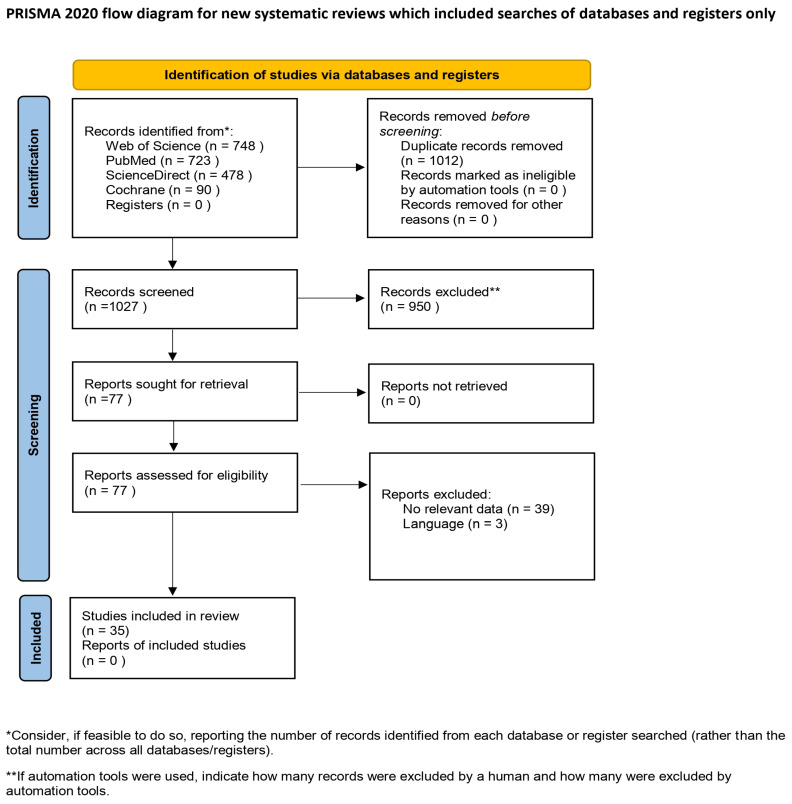
Flow diagram [16].

To identify all of the essential studies that reported relevant information and data concerning surgical technique, femoral attachment, tibial attachment, graft type, fixation method, knee angle during fixation, and graft tension at fixation in LET, an extensive search of the major and significant electronic databases (PubMed, Cochrane Central, ScienceDirect, Web of Science, Embase) was performed by three independent authors (initials blinded for review). Two independent reviewers screened titles and abstracts for eligibility. Three independent reviewers performed full-text review and data extraction. Discrepancies were resolved through discussion and, if necessary, adjudicated by a senior reviewer. The complete Boolean search strings for each database were performed between 1 November and 15 November 2023 in Poland. No restrictions on publication date or language were applied. A systematic investigation was conducted using combinations of the following key terms: (extra-articular OR extraarticular) AND (tenodesis OR plasty OR augmentation OR procedure or reconstruction OR reconstructive OR surgical OR surgery OR technique) AND (ACL OR anterior cruciate ligament). Moreover, an additional intensive search through the references of all identified studies was conducted. To avoid double-counting of data, studies with overlapping populations were identified through careful cross-checking. When duplicates were found, the most comprehensive or recent report was included. In addition to electronic database searching, reference lists of included articles were manually screened for further relevant studies. Formal grey literature databases were not searched, which we acknowledge as a limitation. A systematic review of the collected literature was carried out according to the guidelines of Preferred Reporting Items for Systematic Reviews and Meta-Analyses (PRISMA) (Supplementary Material). The PRISMA checklist of our project was presented in Figure 1. Registration of this systematic review was performed in 2023 using the PROSPERO International Prospective Register of Systematic Reviews (registration number CRD42023428461).

### 2.2. Eligibility Assessment

Screening of databases was carried out independently by two authors (initials blinded for review). After database search, three independent reviewers (initials blinded for review) screened all the papers, identified a title, abstract, and full text concerning the following primary outcomes: indications for surgery, surgical technique, graft type, fixation method, and tibial fixation location, and the following secondary outcomes: demographic data, femoral attachment, tibial attachment, knee angle during fixation, and graft tension at fixation in ACL reconstruction. Inclusion criteria were the following: clinical human studies, level of evidence I–IV, English language. Exclusion criteria were the following: any clinical outcomes and basic science studies in any joint other than the knee, anatomic and radiographic studies, animal studies, editorial articles, surveys, non-English language studies, case studies, reviews, letters to editors, conference abstracts, studies containing incomplete or irrelevant data, and papers without clearly described indications. The senior two authors and experts in evidence-based medicine (initials blinded for review) made the final decision in case of disagreement among the authors.

### 2.3. Data Extraction

Three independent reviewers (initials blinded for review) extracted the initially screened and relevant data, including year of the study, country, type of the study, number of subjects, mean age, gender, indications for surgery, surgical technique, femoral attachment, tibial attachment, graft type, fixation method, tibial fixation location, knee angle during fixation, and graft tension at fixation in ACL reconstruction.

### 2.4. Risk of Bias Assessment

The risk of bias assessment was performed using Version 2 of the Cochrane risk-of-bias tool for randomized trials (RoB 2). Risk of bias was assigned to the following domains as ‘low’, ‘high’, or ‘unclear’: sequence generation/allocation concealment (selection bias), blinding of participants and personnel (performance bias), blinding of outcome assessment (detection bias), incomplete outcome data (attrition bias), selective outcome reporting (reporting bias), and other sources of bias. The quality of papers was assessed independently by three reviewers, with agreement.

## 3. Results

Thirty-five articles published between 1996 and 2023 were analyzed in this systematic review (Figure 1).

The general characteristics and demographic data are presented in Table 1.

The majority of papers were from Italy (n = 11), USA (n = 3), France (n = 3), and Canada (n = 3). Considering all articles, the number of subjects analyzed was 6253, including 4299 men and 1954 women. In four papers, only men participated in the study, and in two, only women. The average age of the subjects was 25.4 years, where in some cases, including the publications of Grassi A et al. and Ventura A et al., average age was given for a particular subgroup, so it had to be recalculated [49,52]. Most of the papers, namely 26, were retrospective, while nine were prospective. The most frequent papers were level 4 evidence (n = 16), less frequently level 3 (n = 12), and seven papers with level 1 evidence.

### 3.1. Surgical Technique

Table 2 contains data regarding surgical technique, which is the name of the operative method used by the author.

Based on it, further, more detailed division criteria were created. Of the 35 papers reviewed, seven surgical techniques of Lateral Extra-articular Tenodesis differing in the way the procedure was performed were separated. These are Lemaire, Lemaire modified, MacIntosh, MacIntosh modified by Coker–Arnold, Marcacci, Ellison modified, and Christel. These methods comprised the vast majority, as 17 papers used the Lemaire modified procedure and 10 papers used the MacIntosh modified by Coker–Arnold approach, making them the most commonly employed. The next methods were in the minority, as the authors used the MacIntosh technique three times. Two papers described the use of the Marcacci method and another two Elison modified. Trichine et al. used the approach described by Christel, while Oni et al. applied the Lemaire method without modification [19,39]. One paper reported the use of two treatment techniques, both modified Lemaire and MacIntosh modified by Coker–Arnold which were compared by Declercq et al. [30].

### 3.2. Femoral Attachment

Most of the papers mentioned fixation location on the lateral distal part of the femur including six articles referring directly to lateral femoral epicondyle (Table 2). Four articles likewise referred to the lateral femoral epicondyle but positioned the attachment slightly proximally and posteriorly. In five cases, authors reported fixation of the graft to the lateral femoral condyle. A significant number of papers, as many as eight, had no information at all regarding the location of the graft placement on the femur. Six authors mentioned the iliotibial band as the graft attachment, and this was mainly related to the MacIntosh and MacIntosh modified by Coker–Arnold techniques. The others, however, determined the fixation site through the nearest anatomical structures such as the distal ridge of Kaplan’s fibers mentioned by El-Azab et al., the insertion of gastrocnemius mentioned by Hantouly et al, or the femoral collateral ligament used for localization mentioned by Jacquet et al. and Getgood et al. [18,20,32,36,53].

### 3.3. Tibial Attachment

Another aspect of surgical techniques analyzed, also relating to the fixation site, was tibial attachment. Most of the authors were unanimous on this point, as in as many as 25 papers, they referred to the tibial attachment as Gerdy’s tubercle (Table 2). That is to say, a nodule located on the lateral and proximal part of the tibia just below the knee joint, which is the attachment area of the iliotibial band. The rest of the authors did not specifically identify the graft attachment site on the tibia.

### 3.4. Fixation Method

The types of fixations in the surgical techniques of the collected papers were Sutures, Staples, Anchor, Interference screw, K-wire, Bioabsorbable Screw and Titanium Screw with a serrated polyethylene washer. Nineteen authors used fixation with Sutures, among them, nine applied the MacIntosh modified by Coker–Arnold technique. Eight publications utilized Staples, where six of the surgeries were performed with the Lemaire modified method (Table 2). In the same method, five authors employed Interference Screws for fixation. Trichine et al. also relied on the Interference Screw [19]. Anchors were used by four authors. K-wire, Bioabsorbable Screws, and Titanium Screws with a serrated polyethylene washer were exclusively applied once. In three articles, there was no information regarding the method of fixing the graft.

### 3.5. Knee Angle During Fixation

Selected papers report various angles of the knee during fixation (Table 2). Nine of the authors did not precisely identify the exact angle. Seven analyzed articles mention flexion between 60° and 70°, five of these procedures were performed using the Modified Lemaire method, the other, used techniques were Marcacci and Ellison. The knee was positioned at 90° of flexion in three of the reviewed papers, using the Modified Lemaire, Marcacci, and MacIntosh modified by Coker–Arnold techniques. The angle of 30° flexion was mentioned by three authors, all of them using the modified Lemaire technique. Also the angle of 20° flexion appears in three papers, two of which report using the modified Lemaire technique; the other used technique was MacIntosh modified by Coker–Arnold. A 45° knee flexion was reported twice, both times while using the modified Lemaire technique. A 90° flexion and unspecified external rotation can be found in two of the papers; both reported using the MacIntosh modified by Coker–Arnold technique. Declercq et al. mention 30° flexion and maximal external rotation while using the MacIntosh modified by Coker–Arnold technique. Mahmoud et al. report a 50° flexion while using the MacIntosh technique [51]. The same technique, but with the knee positioned at 60° flexion and 5° external rotation was used by Christodoulou et al. [41]. The knee positioned at 60° flexion, maximal external rotation, and usage of the Lemaire was reported by Oni et al. [39]. In addition, 90° flexion and 10° or 30° external rotation were reported by Trichine et al. and Alessio-Mazzola et al., respectively; the first one using Christel, the other one the MacIntosh modified by Coker–Arnold technique [19,40]. Guy et al. performed the procedure using the modified Lemaire technique with the knee at full extension [43].

### 3.6. Graft Type

An important point where there was a significant disagreement among authors was the graft type category (Table 3).

In 29 papers, a fragment of iliotibial band (ITB) was used as a graft; in six papers, the type was not specified; meanwhile, Grassi et al. used semitendinosus and gracilis strands [46]. A total of 15 different types of transplants were applied, varying in length or width. In the modified Lemaire method, the most common graft used was the ITB with a length of 8 cm and a width of 1 cm, mentioned by eight writers. Three authors in the MacIntosh modified by Coker–Arnold technique utilized a 10 cm × 1 cm ITB strip. Also, three publishers used a graft specified only as ITB, and another two ITB 6–8 cm × 0.6–0.8 cm. A 13 cm × 1 cm transplant was applied by Viglietta et al. and Monaco et al. [27,31]. The remaining grafts were used once by the authors.

### 3.7. Graft Tension

Twenty-eight authors did not specify the graft tension (Table 3). Minimal tension was applied twice, both while using the modified Lemaire technique. In one of the papers, the graft was taut, but using tension below 20 N, while two mention moderate manual tension of approx. 20 N, all while using the modified Lemaire technique. High manual tension was reported twice, using the MacIntosh modified by Coker–Arnold and Christel technique. Mahmoud et al. report positioning the graft with physiological tension while using the MacIntosh technique [51].

### 3.8. Summary of Key Patterns and Variations

Across the 35 included studies, several areas of strong consensus and significant heterogeneity were observed. The modified Lemaire technique emerged as the most frequently used surgical method (n = 17), followed by the MacIntosh modified by Coker–Arnold technique (n = 10), suggesting a clear preference for these two approaches. Similarly, Gerdy’s tubercle was consistently reported as the tibial attachment site in 25 studies, indicating strong anatomical consensus. In contrast, the femoral attachment site showed considerable variation, with placements reported at the lateral femoral epicondyle, lateral condyle, and several anatomical landmarks, and eight studies provided no clear information.

Iliotibial band grafts were the most common type used (n = 29), yet graft length and width varied notably, as did the degree of graft tension—with only seven studies specifying this parameter. The knee angle during fixation was another area of high heterogeneity, with values ranging from full extension to 90° flexion and inconsistent use of external rotation.

Clinically, these variations may impact graft isometry, rotational control, and long-term joint stability. The predominance of certain techniques suggests emerging standards in practice, while inconsistencies in fixation parameters highlight the need for further biomechanical and clinical outcome studies to determine optimal protocols.

## 4. Discussion

Our study findings showed that the main frequent and repeated surgical procedure for LET was the Lemaire modified method. The results of our investigation also demonstrated that the iliotibial band was the most commonly used and repeated graft. Moreover, Gerdy’s tubercle was the only site of tibial fixation. As far as we know, this is the first systematic review which deals with the systematization of various LET techniques across the years. Due to the growing interest in LET procedures in recent years, it appears crucial to systematize and to establish relevant technique for this procedure.

This systematic review highlights the modified Lemaire and MacIntosh (Arnold–Coker modification) techniques as the most frequently reported and widely used procedures for Lateral Extra-articular Tenodesis. The iliotibial band remains the predominant graft choice, with consistent tibial fixation at Gerdy’s tubercle. Despite these patterns, significant variations in technical execution—particularly regarding femoral attachment, fixation angles, and graft tensioning—limit direct comparison across studies and challenge efforts to identify an optimal surgical technique.

The incomplete and inconsistent reporting of key procedural details further constrain the reliability and generalizability of current evidence. To advance the field, future research should focus on standardized descriptions of surgical methods, comparative studies of technique-specific outcomes, and clear protocols for graft fixation parameters. Establishing these standards is essential for improving reproducibility, optimizing clinical outcomes, and guiding surgical decision-making in patients undergoing LET, particularly when combined with ACL reconstruction.

The history of LET is complex. In 1989, the American Orthopedic Society of Sports Medicine (AOSSM) published an opinion that LET procedures were not beneficial due to the potential for postoperative stiffness and the development of knee osteoarthritis [54]. The return of the LET procedure is related to its use as an isolated procedure for older patients in case of contraindications to ACLR [55]. The improved understanding of the anterolateral complex of the knee’s role as a stabilizing component, particularly its defense against excessive internal rotation, is also essential to the resurgence of LET [56]. The anatomical structures of the anterolateral complex responsible for rotational stability are the superficial and deep fibers of the ITB and the ALL, but the coordinated biomechanics of these structures that function in the control of rotational laxity are unknown [57]. Noyes et al. described the role of ALL and ITB in knee rotational stability. The results of their study showed that the ITB acts as a secondary limitation of tibial translation and internal rotation, and the ALL and ITB limit internal rotation at high flexion angles. As a result, an ALC injury places significant stress on the ACL, especially in sports that involve cutting, jumping, and pivoting [58]; however, for elite athletes who expect an extremely high level of physical performance, current ACL reconstruction techniques produce variable results [59]. For this reason, it has been proposed to combine ACLR with LET in a selected group of patients. The addition of primary LET to ACLR improves control of rotational laxity over time without increasing the rate of complications [60]. According to Getgood et al. the indications for combining LET with ACLR are revision ACLR, high-grade rotational laxity (a grade-2 or -3 pivot shift), generalized ligamentous laxity, or genu recurvatum of >10° and ACL injury in a young patient (<25 years old) returning to a contact pivoting sport [61]. Authors of recent research publications have performed LET procedures with various techniques, using different graft sizes, locations, and methods of fixation, such as Screws or Sutures. In this article, however, special attention was paid to the type of technique used and its detailed aspects. Indeed, the variety of techniques used to perform LET is quite large; in our study, seven of them were presented, but Slette et al., in their systematic review, described as many as 12 [62].

The aim of this study was to identify the most frequently employed lateral LET techniques and to describe their technical characteristics. The anatomical location of graft attachment on both the femur and tibia, as well as the method and angle of fixation, were evaluated. Our findings demonstrate that the modified Lemaire technique and the MacIntosh technique modified by Coker–Arnold predominate in current practice, representing over 75% of the reviewed studies. This predominance is consistent with recent biomechanical data indicating that these methods offer superior control of anterolateral rotational laxity, resulting in significantly lower ipsilateral ACL rupture rates and reduced pivot-shift occurrences compared with alternative techniques [63].

Nonetheless, the continued use of other methods—including the Marcacci, modified Ellison, and Christel techniques—underscores the absence of a universal consensus regarding the “ideal” LET procedure. While the tibial attachment site was consistent across studies, greater variation was observed in the femoral attachment site. Reported positions ranged from the lateral femoral epicondyle to locations determined by adjacent anatomical landmarks such as Kaplan’s fibers or the gastrocnemius tendon insertion. These differences likely reflect both surgeon preference and variations in anatomical interpretation of the anterolateral complex. Given the biomechanical importance of graft isometry and the need to avoid over-constraint, the lack of standardization in femoral fixation techniques warrants further investigation. This is of particular relevance in light of reports suggesting that excessive lateral compartment constraint may predispose to the development of osteoarthritis [64]. Similarly, the knee flexion angle during fixation demonstrated considerable variability, ranging from full extension to 90° of flexion. This parameter directly influences graft tensioning and postoperative kinematics, yet more than one-quarter of studies failed to report the fixation angle. This lack of documentation impedes reproducibility and hinders accurate comparison of clinical outcomes. Graft tension at the time of fixation was also poorly reported, with 80% of studies omitting this detail. Among those that did, reported values ranged from minimal manual tension to >20 N, reflecting the absence of standardized protocols. Given that excessive tensioning risks over-constraint and insufficient tensioning risks persistent instability, this represents a critical gap in both reproducibility and outcome predictability. This article also summarizes various graft dimensions; however, for the Lemaire technique, a length of approximately 8 cm and a width of 1 cm appears optimal. Nikolaos E. et al. [65] identified the principal advantage of the modified Lemaire technique as its technical simplicity. It has no steep learning curve and can be readily performed by surgeons already experienced in ACL reconstruction. In addition to its simplicity, the technique is versatile and can be adapted to individual surgeon preferences and patient-specific anatomical considerations, as evidenced by the numerous variations in graft attachment described in the literature.

### 4.1. The Lemaire Procedure

This literature review indicates that the most commonly used LET procedure is the modified Lemaire technique (MLT). Lemaire described his LET technique in 1967, in an article in which he noted that poor outcomes following ACL injury were related to rotational instability. He considered controlling this instability crucial to controlling anterior translation. The original method developed by Lemaire was based on the use of a loop made of a strip of fascia, passed through a bone tunnel [53]. Apart from Lemaire, who modified and improved his surgical technique throughout the 1970s and 1980s, two French researchers, Christel and Dijah, modified Lemaire’s original technique by using an 180° inverted, short strip of ITB. The authors noted that the advantages of the introduced modification were better graft isometrics, shorter skin incision, and shorter graft harvesting [66]. Currently, the most commonly used modified Lemaire technique is the procedure described by Jesani et al. [14]. This technique is characterized by a much shorter skin incision and the use of a significantly smaller graft. It is still a technique using a loop passed deep to the FCL, but using modern methods of fixing it to the femur [67]. In the modification of the Lemaire method described by Jesani and Getgood, an autologous fascia graft is used in the form of an approximately 8 cm long and 1 cm wide strip taken from the posterior half of the ITB. The graft is carried under the FCL from distal to proximal and anchored slightly anteriorly and proximally to the lateral head of the gastrocnemius tendon in the lateral part of the lateral femoral condyle. The graft tension and fixation in the femur is performed in 60° of knee flexion and in neutral rotation of the foot, which protects against lateral compartment over-constraint [14]. A similar technique was proposed by Schlichte et al., but the procedure assumes that the graft is tensioned in 30° of knee flexion. Moreover, the work of Schlichte et al. concerns the pediatric population [68]. Mostly, very good MLT results are observed. Mechanical studies indicate a significant reduction in rotatory knee laxity assessed by the pivot shift test [69]. Clinical outcomes of LET utilizing MLT combined with ACLR are good in a high-risk group of patients with risk factors such as high-risk sports, ligamentous laxity, knee recurvatum exceeding 10°, and high-grade pivot shift (grade two or higher) [50,70]. Available data suggest that the combination of LET with ACLR in high-risk patients protects against graft rupture and this is probably the biggest benefit of this procedure [15,33,43]. Apart from patients at risk, indications for combining LET with ACLR include patients with lateral coronal plane laxity, increased posterior tibial slope, concomitant lateral meniscal deficiency, and anterolateral capsular injury confirmed by MRI [5]. Combining LET with ACLR is contraindicated in knees with ACL insufficiency with concomitant posterolateral corner injury (PLCI) or PLCI laxity, in cases of lateral compartment knee osteoarthritis, and in skeletally immature patients due to risk of injury to the femoral physis [5,68,71].

### 4.2. The MacIntosh Procedure

The results of MacIntosh’s original technique were published in 1985 [72]. This method uses a 25 × 4 cm ITB graft that is pulled under the LCL from a distal to a proximal direction. It is then guided through a subperiosteal tunnel to the lateral intermuscular septum (LIS). After leaving the LIS, the graft is passed around the lateral femoral condyle to the knee joint and then through the tibial tunnel back to the attachment on Gerdy’s tubercle, where it is fixed [5]. The most commonly used modification of the original MacIntosh method is the one modified by Arnold–Coker. This procedure ranked second among treatment method preferences. In this technique, a graft of about 8–12 cm in length and about 1 cm in width is taken from the central part of the ITB. After cutting off in the proximal part, the graft is sewn with a Krakow suture over a distance of approximately 1 cm and then pulled deep under the FCL. Then, the suture is pulled deep into the intermuscular septum, creates a loop, and is secured with a suture, connecting the band to itself. The suture is tightened in 30° of knee flexion and neutral rotation of the foot [73]. The main advantage of the described method is the lack of need for creating a bone tunnel that could connect with the femoral tunnel created during ACLR [73]. Viglietta et al. conducted research comparing the risk of knee arthrosis after using isolated ACLR (iACLR) and the combined ACLR and LET procedure according to MacIntosh in the Arnold–Coker modification (MACM). The authors showed that the long-term risk of arthrosis is higher in the iACLR [27]. Also, Declerq et al. described MACM as a safe procedure that does not increase the risk of osteoarthritis in the lateral compartment [30]. MACM is considered a procedure that reduces the rotational instability of the knee [18,24,40,42,49] and significantly lowers the risk of graft rupture [27,31].

### 4.3. The Other Procedures

Some authors described other, less common surgical techniques in their papers, such as Lemaire [39], Marcacci [28,52], MacIntosh [29,41,51], modified Ellison [26,34], and Christel [66]. Some authors believe that the MacIntosh procedure effectively restores joint stability [29,41,51]. The modified Ellison method was deemed safe by Feller et al. in a cohort with a low graft rupture rate and a high risk of re-injury [26]. Marcacci et al. and their original technique show maintenance of knee stability at long-term follow-up despite a lack of impact on knee osteoarthritis [28]. Grassi et al. reported that using this technique decreased the readmission rate after surgery, including knee stiffness, swelling, and infection [52]. Even with these promising results, further current studies are required before selecting any of these methods over MLT or MACM.

Declerq et al. compared both techniques, the MACM and the MLT, and found no statistically significant differences between both techniques in mean postoperative: IKDC, Lysholm, and Tefner score. Despite some data regarding the LET with ACLR were conflicting [22,32,36], and regardless of the variety of the modifications, many authors suggested that LET combined with ACLR is a safe technique and should be considered as a concomitant due to the postoperative positive outcomes [17,23,29,30,35,37,43].

One major limitation of this systematic review lies in the inconsistent and often incomplete reporting within the analyzed studies. Rather than simply reflecting a lack of available data, this highlights a deeper issue related to study quality and methodological rigor. Key surgical details—such as graft attachment points, fixation methods, knee flexion angle, and procedural modifications—were frequently omitted or only superficially described. For example, techniques like the modified Lemaire procedure were often cited without clear documentation of how they differed from the original, or who introduced the modifications. These gaps significantly limited the comparability of surgical techniques across studies.

The overall quality of the evidence base was moderate to low. A substantial proportion of included studies were retrospective and classified as Level 3 or 4 evidence. This limited the strength of conclusions that could be drawn and increased the risk of selection and reporting biases. While a formal risk of bias assessment was conducted, the influence of study design on the findings is important to acknowledge; retrospective studies, for example, were less likely to provide detailed technical descriptions or standardized outcome measures, reducing the reliability of technical comparisons.

Furthermore, technique preferences may have been influenced by evidence level and study design. Higher-quality, prospective studies tended to focus on the most widely accepted procedures—such as the modified Lemaire and modified MacIntosh techniques—while less common or experimental techniques were more frequently described in retrospective case series or low-sample studies. As a result, the dominance of certain techniques in this review may reflect the availability and quality of published data rather than definitive clinical superiority.

Temporal, language, and publication biases also likely impacted the findings. Techniques that have evolved or fallen out of favor may be underrepresented due to limited recent publication. Only articles in selected languages and indexed in major databases were included, potentially excluding relevant studies or alternative techniques such as Benum’s approach.

Lastly, the pattern of missing data—particularly regarding fixation angles, graft tensioning, and follow-up duration—further undermines the reproducibility and generalizability of the findings. These technical details are crucial for surgical replication and outcome interpretation. The lack of standardization across studies not only limits evidence-based recommendations but also makes it difficult to draw robust conclusions about optimal surgical technique.

Future studies should aim for higher methodological rigor, including prospective design, standardized surgical reporting, and consistent outcome measures, to strengthen the evidence base and support clearer clinical recommendations.

## 5. Conclusions

Lateral Extra-articular Tenodesis (LET) is an isolated extra-articular procedure originally developed to address rotational instability in anterior cruciate ligament (ACL) injuries. Although it lost prominence with the rise of arthroscopic techniques and was largely abandoned for decades, recent evidence has sparked renewed interest in its use. Contemporary studies have highlighted LET as a valuable adjunct in ACL reconstruction, particularly for patients exhibiting high-grade instability.

In our study, we examined the various modifications of the LET technique currently in use. Despite the diversity in surgical approaches, the Lemaire-modified technique emerged as the most commonly adopted variation. The most frequently utilized graft was the iliotibial band, with Sutures being the predominant method of fixation. Tibial fixation was consistently located at Gerdy’s tubercle, while the femoral attachment site showed variability, being either the lateral femoral condyle or the lateral femoral epicondyle. This variability underscores the need for further research to determine the optimal femoral fixation point.

Although once forgotten, the LET technique is now steadily regaining traction and acceptance in clinical practice. Current data support its growing role as a complementary procedure in ACL surgery, emphasizing its importance in managing complex cases with persistent knee instability.

## Figures and Tables

**Table 1:** The general characteristics and demographic data of the included studies on LET technique [17,18,19,20,21,22,23,24,25,26,27,28,29,30,31,32,33,34,35,36,37,38,39,40,41,42,43,44,45,46,47,48,49,50,51].

Author	Year	Country	Study Design	Level of Evidence	Type of Study	Number of Subjects	Male	Female	Mean Age
Green et al. [17]	2023	USA	Case series	4	Retrospective	48	27	21	14.2
Hantouly et al. [18]	2023	Qatar	Cohort study	3	Retrospective	100	94	6	28.15
Trichine et al. [19]	2013	Algieria	Randomized trial	1	Prospective	120	120	0	28.15
Mahmoud et al. [51]	2021	Australia	Matched cohort study	3	Retrospective	72	55	17	25
El-Azab et al. [20]	2023	Egypt, Austria	Randomized Comparative Study	1	Prospective	100	73	27	27.5
Joseph et al. [21]	2020	France, Switzerland	Comparative study	3	Retrospective	87	57	30	29.7
Eggeling et al. [22]	2021	Germany	Cohort study	3	Retrospective	78	48	30	28.7
Monyart et al. [23]	2023	Spain	Case series	4	Prospective	46	31	15	36.3
Vadalà et al. [24]	2012	Italy	Comparative study	3	Prospective	60	0	60	27
Gibbs et al. [25]	2021	USA, Japan	Cohort study	3	Retrospective	20	12	8	20.8
Feller et al. [26]	2021	Australia	Case series	4	Retrospective	25	21	4	8.5
Viglietta et al. [27]	2021	Italy	Cohort study	3	Retrospective	164	126	38	27.3
Marcacci et al. [28]	2009	Italy	Case series	4	Retrospective	60	45	15	-
Ibrahim et al. [29]	1999	Kuwait	Case series	4	Retrospective	153	153	0	23.7
Declercq et al. [30]	2021/22	Belgium	Case series	4	Retrospective	86	69	17	26.1
Monaco et al. [31]	2022	Italy	Cohort study	3	Retrospective	111	69	42	16.2
Getgood et al. [32]	2020	Canada	Randomized Controlled Trial	1	Retrospective	356	154	202	18.9
Heard et al. [33]	2023	Canada	Randomized Clinical Trial	1	Prospective	618	302	316	18.9
Farinelli et al. [34]	2023	Austria	Case series	4	Retrospective	27	27	0	23.15
Alm et al. [35]	2020	Germany	Case series	4	Retrospective	73	39	34	31
Jacquet et al. [36]	2021	France	Cohort study	3	Retrospective	266	190	76	30.4
Keizer et al. [37]	2022	Netherlands	Cohort study	3	Retrospective	78	57	21	29.3
Borim et al. [38]	2023	Spain	Case series	4	Prospective	19	9	10	27.7
Oni & Crowder [39]	1996	UK	Randomized Controlled Trial	1	Prospective	32	26	6	-
Alessio-Mazzola et al. [40]	2019	Italy	Case series	4	Retrospective	24	24	0	23.8
Christodoulou et al. [41]	2005	Greece	Case series	4	Retrospective	52	47	5	41
Legnani et al. [42]	2022	Italy	Case series	4	Retrospective	36	22	14	27.6
Guy et al. [43]	2022	France	Cohort study	3	Retrospective	81	45	36	22.5
Legnani et al. [44]	2019	Italy	Case series	4	Retrospective	9	7	2	23.3
Zanna et al. [45]	2023	Italy	Case series	4	Retrospective	17	14	3	26.4
Grassi et al. [46]	2021	Italy	Case series	4	Retrospective	2559	2009	550	30.9
Guzzini et al. [47]	2016	Italy	Case series	4	Retrospective	16	0	16	26.4
Chiba et al. [48]	2021	USA	Randomized Controlled Trial	1	Prospective	18	11	7	20.45
Ventura et al. [49]	2021	Italy	Case series	3	Retrospective	24	19	5	30.35
Getgood et al. [50]	2020	Canada	Randomized Controlled Trial	1	Prospective	618	297	321	18.9

**Table 2:** The general characteristics of surgical technique [17,18,19,20,21,22,23,24,25,26,27,28,29,30,31,32,33,34,35,36,37,38,39,40,41,42,43,44,45,46,47,48,49,50,51].

Author	Type of Technique	Femoral Attachment	Tibial Attachment	Fixation Method	Knee Angle During Fixation
Green et al. [17]	modified Lemaire	proximal and posterior to the lateral femoral epicondyle	Gerdy’s Tubercle	Sutures	30° flexion
Hantouly et al. [18]	modified Lemaire	3 cm higher to gastrocnemius insertion in the posterior third of the femur	Gerdy’s Tubercle	Sutures/Staples/Anchor	30° flexion
Trichine et al. [19]	Christel	Lateral femoral condyle	Gerdy’s Tubercle	Interference screw	90° flexion, 10° external rotation
Mahmoud et al. [51]	MacIntosh	ITB	Gerdy’s Tubercle	Sutures	50° flexion
El-Azab et al. [20]	modified Lemaire	distal ridge of Kaplan’s fibers	n/a	Interference screw	n/a
Joseph et al. [21]	modified Lemaire	n/a	Gerdy’s Tubercle	Interference screw	
Eggeling et al. [22]	Modified Lemaire	1 cm proximal and posterior to the lateral epicondyle	Gerdy’s Tubercle	Interference screw	45° flexion
Monyart et al. [23]	modified Lemaire	n/a	n/a	n/a	n/a
Vadalà et al. [24]	Macintosh modified by Coker–Arnold	n/a	Gerdy’s Tubercle	Sutures	n/a
Gibbs et al. [25]	modified Lemaire	proximal and posterior to the lateral femoral epicondyle	Gerdy’s Tubercle	Staples	Between 60° and 70° flexion
Feller et al. [26]	modified Ellison	ITT strip passed deep to the LCL from proximal to distal and reattached to Gerdy’s tubercle	Gerdy’s Tubercle	Sutures/Anchor	Between 60° and 70° flexion
Viglietta et al. [27]	MacIntosh modified by Coker–Arnold	n/a	Gerdy’s Tubercle	Sutures	90° flexion, external rotation
Marcacci et al. [28]	Marcacci	Lateral femoral condyle	Gerdy’s Tubercle	Staples	90° flexion
Ibrahim et al. [29]	MacIntosh	n/a	Gerdy’s Tubercle	Sutures	90° flexion
Declercq et al. [30]	MacIntosh modified by Coker–Arnold/modified Lemaire	ITB	Gerdy’s Tubercle	Sutures/Anchor	30° flexion, maximal external rotation
Monaco et al. [31]	MacIntosh modified by Coker–Arnold	ITB	Gerdy’s Tubercle	Sutures	90° flexion, external rotation
Getgood et al. [32]	modified Lemaire	Distal femur anteriorly to the intermuscular septum and proximally to the femoral attachment site of the FCL	Gerdy’s Tubercle	Staples	Between 60° and 70° flexion
Heard et al. [33]	modified Lemaire	n/a	n/a	Sutures	Between 60° and 70° flexion
Farinelli et al. [34]	modified Ellison	ITT strip passed deep to the LCL from proximal to distal and reattached to Gerdy’s tubercle	Gerdy’s Tubercle	Sutures/Anchor	n/a
Alm et al. [35]	modified Lemaire	1 cm proximal and posterior to the lateral epicondyle	Gerdy’s Tubercle	Sutures/K-wire	45° flexion
Jacquet et al. [36]	modified Lemaire	Distal femur anteriorly to the intermuscular septum and proximally to the femoral attachment site of the FCL	Gerdy’s Tubercle	Interference screw	20° flexion
Keizer et al. [37]	modified Lemaire	n/a	n/a	Interference screw	20° flexion
Borim et al. [38]	modified Lemaire	n/a	n/a	n/a	n/a
Oni & Crowder [39]	Lemaire	Lateral femoral condyle	n/a	Sutures	60° flexion, maximal external rotation
Alessio-Mazzola et al. [40]	MacIntosh modified by Coker–Arnold	ITB	Gerdy’s Tubercle	Sutures	90° flexion, 30° external rotation
Christodoulou et al. [41]	MacIntosh	Lateral femoral epicondyle	Gerdy’s Tubercle	Titanium screw with a serrated polyethylene washer	60° flexion, 5° external rotation
Legnani et al. [42]	MacIntosh modified by Coker–Arnold	ITB	n/a	Sutures	n/a
Guy et al. [43]	modified Lemaire	Modified Lemaire fixation	n/a	Bioabsorbable screw	Full extension
Legnani et al. [44]	MacIntosh modified by Coker–Arnold	Lateral femoral epicondyle	Gerdy’s Tubercle	Sutures	n/a
Zanna et al. [45]	MacIntosh modified by Coker–Arnold	Lateral femoral epicondyle	Gerdy’s Tubercle	Sutures	90° flexion
Grassi et al. [46]	Marcacci	Lateral femoral condyle	Gerdy s Tubercle	Staples	Between 60° and 70° flexion
Guzzini et al. [47]	MacIntosh modified by Coker–Arnold	Lateral femoral epicondyle	Gerdy s Tubercle	Sutures	n/a
Chiba et al. [48]	modified Lemaire	Lateral femoral epicondyle	Gerdy’s Tubercle	Staples	Between 60° and 70° flexion
Ventura et al. [49]	MacIntosh modified by Coker–Arnold	ITB	n/a	Sutures	20° flexion
Getgood et al. [50]	modified Lemaire	Lateral femoral condyle	n/a	Staples	Between 60° and 70° flexion

**Table 3:** The occurrence of graft type and its tension during fixation [17,18,19,20,21,22,23,24,25,26,27,28,29,30,31,32,33,34,35,36,37,38,39,40,41,42,43,44,45,46,47,48,49,50,51].

Author	Graft Type	Graft Tension During Fixation
Green et al. [17]	ITB 8 cm × 1 cm	n/a
Hantouly et al. [18]	ITB 10–15 cm × 1 cm	n/a
Trichine et al. [19]	ITB 7.5 cm × 1.2 cm	high manual tension
Mahmoud et al. [51]	ITB 12–15 cm	physiological tension
El-Azab et al. [20]	n/a	n/a
Joseph et al. [21]	ITB 8 cm × 1 cm	n/a
Eggeling et al. [22]	ITB 6–8 cm × 0.6–0.8 cm	n/a
Monyart et al. [23]	n/a	n/a
Vadalà et al. [24]	ITB	n/a
Gibbs et al. [25]	ITB	moderate manual tension approx. 20 N
Feller et al. [26]	ITB	n/a
Viglietta et al. [27]	ITB 13 cm × 1 cm	n/a
Marcacci et al. [28]	n/a	n/a
Ibrahim et al. [29]	ITB 15 cm × 1 cm	n/a
Declercq et al. [30]	ITB 6–8 cm × 1 cm	n/a
Monaco et al. [31]	ITB 13 cm × 1 cm	n/a
Getgood et al. [32]	ITB 8 cm × 1 cm	minimal tension
Heard et al. [33]	ITB 8 cm × 1 cm	taut; <20 N
Farinelli et al. [34]	n/a	n/a
Alm et al. [35]	ITB 6–8 cm × 0.6–0.8 cm	n/a
Jacquet et al. [36]	ITB 8 cm × 1 cm	n/a
Keizer et al. [37]	n/a	n/a
Borim et al. [38]	n/a	n/a
Oni & Crowder [39]	ITB 15 cm × 5 cm made into a tube	n/a
Alessio-Mazzola et al. [40]	ITB 10 cm × 1 cm	n/a
Christodoulou et al. [41]	ITB 8–10 cm × 1.3–1.6 cm	n/a
Legnani et al. [42]	ITB	n/a
Guy et al. [43]	n/a	n/a
Legnani et al. [44]	ITB 8–10 cm × 1 cm	n/a
Zanna et al. [45]	ITB 10 cm × 1 cm	n/a
Grassi et al. [46]	Semitendinosus and gracilis	n/a
Guzzini et al. [47]	ITB 10 cm × 1 cm	n/a
Chiba et al. [48]	ITB 8 cm × 1 cm	moderate manual tension approx. 20 N
Ventura et al. [49]	ITB	high manual tension
Getgood et al. [50]	ITB 8 cm × 1 cm	minimal tension

## Data Availability

All data generated or analyzed during this study are included in this published article.

## Supplementary Materials

PRISMA checklist. The following supporting information can be downloaded at: https://www.mdpi.com/article/10.3390/jcm14186510/s1, PRISMA 2020 Checklist [16].

## Abbreviations

The following abbreviations are used in this manuscript:

ACL	Anterior cruciate ligament
ALC	Anterolateral complex
LCL	Lateral collateral ligament
ACLR	Anterior cruciate ligament reconstruction
LET	Lateral Extra-articular Tenodesis
PLCI	Posterolateral corner injury
LIS	Lateral intermuscular septum
iACLR	Isolated anterior cruciate ligament reconstruction
MACM	Arnold–Coker modification
